# Molecular Characterization of a Novel Big Defensin from Clam *Venerupis philippinarum*

**DOI:** 10.1371/journal.pone.0013480

**Published:** 2010-10-20

**Authors:** Jianmin Zhao, Chenghua Li, Aiqin Chen, Lingyun Li, Xiurong Su, Taiwu Li

**Affiliations:** 1 Yantai Institute of Coastal Zone Research, Chinese Academy of Sciences, Yantai, China; 2 Faculty of Life Science and Biotechnology, Ningbo University, Ningbo, China; 3 College of Animal Science and Technology, Northeast Agriculture University, Harbin, China; University of Poitiers, France

## Abstract

Antimicrobial peptides (AMPs) are important mediators of the primary defense mechanism against microbial invasion. In the present study, a big defensin was identified from *Venerupis philippinarum* haemocytes (denoted as VpBD) by RACE and EST approaches. The VpBD cDNA contained an open reading frame (ORF) of 285 bp encoding a polypeptide of 94 amino acids. The deduce amino acid sequence of VpBD shared the common features of big defensin including disulfide array organization and helix structure, indicating that VpBD should be a new member of the big defensin family. The mRNA transcript of VpBD was up-regulated significantly during the first 24 hr after *Vibrio anguillarum* challenge, which was 7.4-fold increase compared to that of the control group. Then the expression decreased gradually from 24 hr to 96 hr, and the lowest expression level was detected at 96 hr post-infection, which was still 3.9-fold higher than that of control. The mature peptide of VpBD was recombined in *Escherichia coli* and purified for minimum inhibitory concentration (MIC) determination. The rVpBD displayed broad-spectrum inhibitory activity towards all tested bacteria with the highest activity against *Staphyloccocus aureus* and *Pseudomonas putida*. These results indicated that VpBD was involved in the host immune response against bacterial infection and might contribute to the clearance of invading bacteria.

## Introduction

The Manila clam, *Venerupis philippinarum*, is an important marine bivalve for commercial fisheries, accounting for about 80% of mudflat fishery production in China (China Bureau of Fisheries, 2004). In the last decades, clam culture in China is sustaining a severe mortality problem, and suffering great economic losses [1]. Although positive results have come from treatment of antibiotics, increasing concerns of antibiotics use have prompted interests in developing alternative strategies for growth and health management [2], [3], [4]. The gene-encoded cationic antimicrobial peptides (AMPs) are major humoral components of the innate defense systems [5], [6], which were considered as promising therapeutic candidates for their innate advantages of less bacterial resistance and very specific targets [3]. Moreover, the discovery of antimicrobial peptides provides new clues for a fundamental understanding of the species immunity response and for further establishment of disease control in the practice of aquaculture industry.

As cationic and amphiphilic molecules, AMPs showed attractive perspective as substitutes for antibiotics in aquaculture for their biochemical diversity, broad specificity against bacteria, fungi or even virus [6], [7], [8], [9], [10], [11], [12]. In mollusk, the presence of lower molecular mass AMPs started to be investigated from last decade [13]. To date, approximately 20 AMPs have been isolated or cloned from mollusks, mainly from mussels (for review see [14]).

Big defensin is one of the AMPs which possess remarkable microbicidal activity against Gram-positive, Gram-negative bacteria and fungi. Until now, only one molluscan big defensin was identified from bay scallop *Argopecten irradians*. The recombinant AiBD could inhibit the growth of both Gram-positive and Gram-negative bacteria, and also showed strong fungicidal activity towards yeast [15]. However, little information is available about the molecular features and immune response against pathogen infection in the commercially cultured clam *Venerupis philippinarum*. The main objectives of the present study are to: (1) clone the full-length cDNA of big defensin from *V. philippinarum* (VpBD); (2) investigate the expression profile of VpBD post infection of *Vibrio* pathogen; (3) elucidate the antibacterial activity of the recombinant VpBD *in vitro*.

## Results

### cDNA cloning and sequence analysis of VpBD

**Figure 1:**
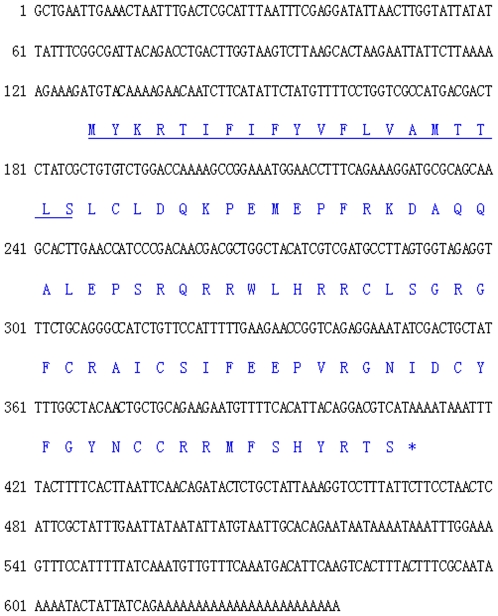
The nucleotide sequence (above) and its deduced amino acid sequence (below) of VpBD. Nucleotides were numbered from the first base at the 5′end. The signal peptide was underlined. The asterisk indicated the stop codon.

A 640 bp fragment representing the complete cDNA of VpBD was obtained by 5′RACE from a cDNA library of *V. philippinarum*. The sequence was deposited in GenBank under accession no. HM562672. The deduced amino acid sequence of VpBD was shown in Fig. 1. The complete sequence of VpBD cDNA contained a 5′ untranslated region (UTR) of 126 bp, a 3′ UTR of 229 bp with a canonical polyA tail, and an open reading frame (ORF) of 285 bp encoded a polypeptide of 94 amino acids with the predicted molecular weight of 11.35 kDa and the theoretical isoelectric point of 9.46. The putative signal peptide was identified at the N-terminal sequence with the cleavage site at amino acid position 20. The mature peptide was analyzed using the antimicrobial peptide predictor program (http://aps.unmc.edu/AP/prediction/prediction_main.php). The results showed that VpBD possessed characteristic features of AMPs including helix structure formation, a net positive charge (+5) and a high percentage of hydrophobic residues (36%).

### Homology analysis of VpBD

**Figure 2:**
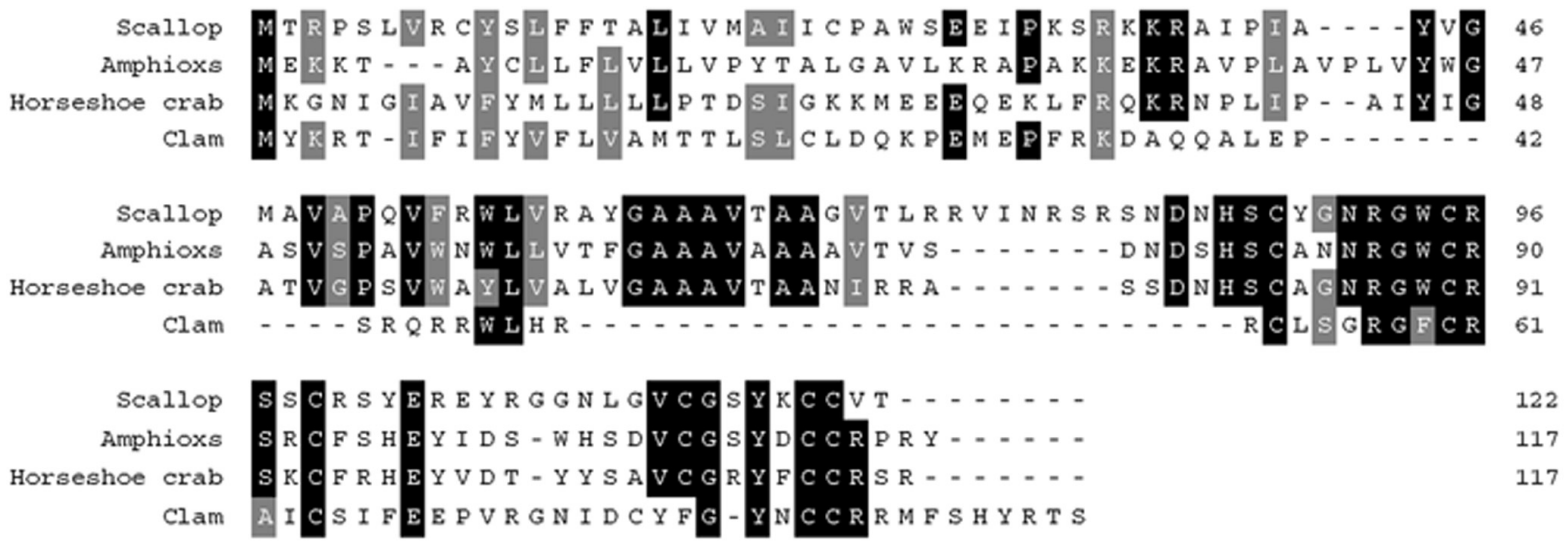
Multiple alignment of VpBD with other known big defensins. Identical amino acids were in white letters with black background, and gray background indicated high levels of amino acid similarity.

The deduced amino acid sequence of VpBD was aligned with other known big defensins by CLUSTALW program and low similarities were found between VpBD and other counterparts (14% –20%). The consensus pattern C-X6-C-X3-C-X13(14)-C-X4-C-C in defensin domain was relatively conserved as typically observed in big defensin from horseshoe crab and bay scallop (Fig. 2). The disulfide array in VpBD was postulated to be identical to that of big defensin from horseshoe crab (C1–C5, C2–C4, and C3–C6).

### The expression profile of VpBD after *Vibrio* challenge

**Figure 3:**
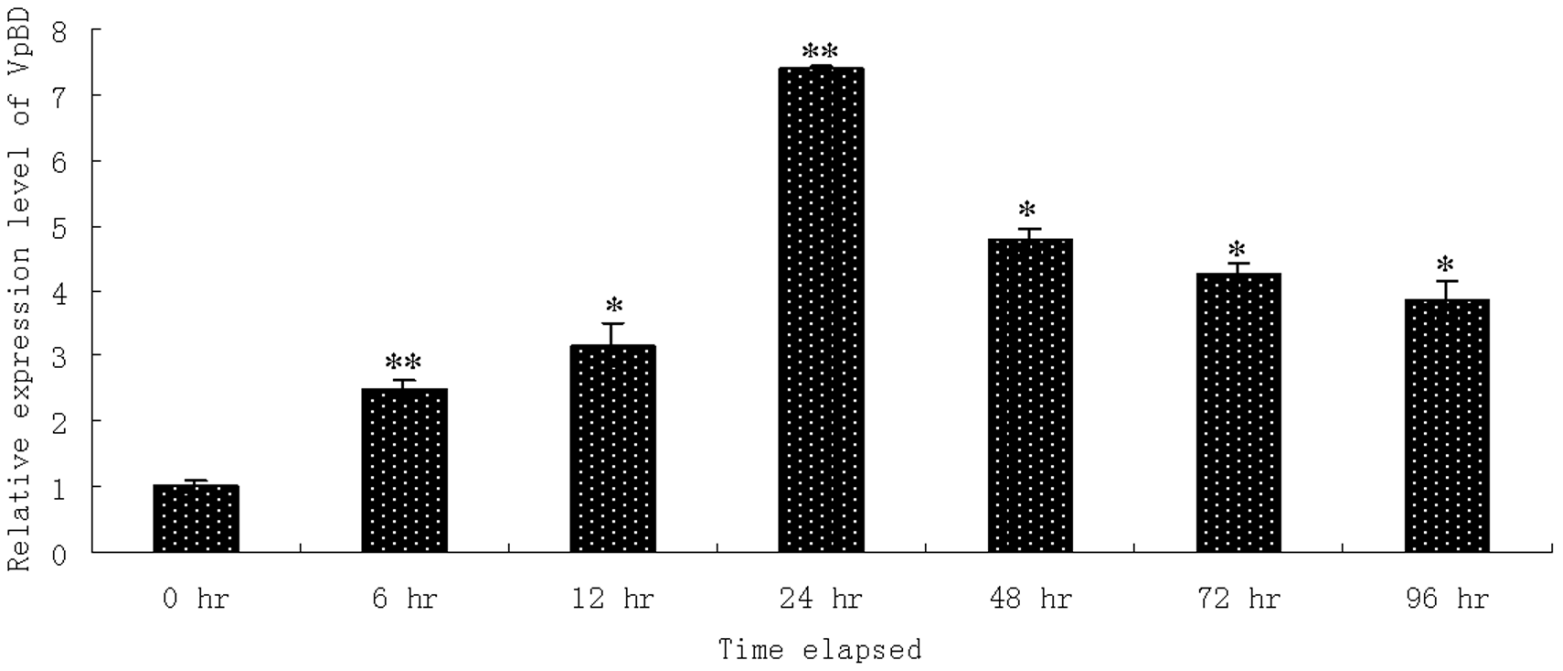
Time-course expression level of VpBD transcript in haemocytes after *Vibrio anguillarum* infection measured by quantitative real-time PCR at 0 hr, 6 hr, 12 hr, 24 hr, 48 hr, 72 hr and 96 hr. Each symbol and vertical bar represented the mean ± S.D (n = 5). Significant differences between challenged group and control group were indicated by an asterisk (*P*<0.05) and two asterisks (*P*<0.01), respectively.

The temporal mRNA expression of VpBD in the haemocytes post-*Vibrio* challenge was shown in Fig. 3. During the first 24 hr after pathogen challenge, the expression level of VpBD mRNA was obviously up-regulated and reached 7.4-fold compared to that of control group. After that, the expression level was decreased gradually from 24 hr to 96 hr, and the lowest expression level was detected at 96 hr post-bacterial infection, which was 3.9-fold higher than that of control group. Significant differences of the expression level of VpBD were observed at 6 hr (*P*<0.01), 12 hr (*P*<0.05), 24 hr (*P*<0.01), 48 hr (*P*<0.05), 72 hr (*P*<0.05) and 96 hr (*P*<0.05) post the challenge of *Vibrio* compared to the control group.

### Characterization of recombinant peptide by electrophoresis

**Figure 4:**
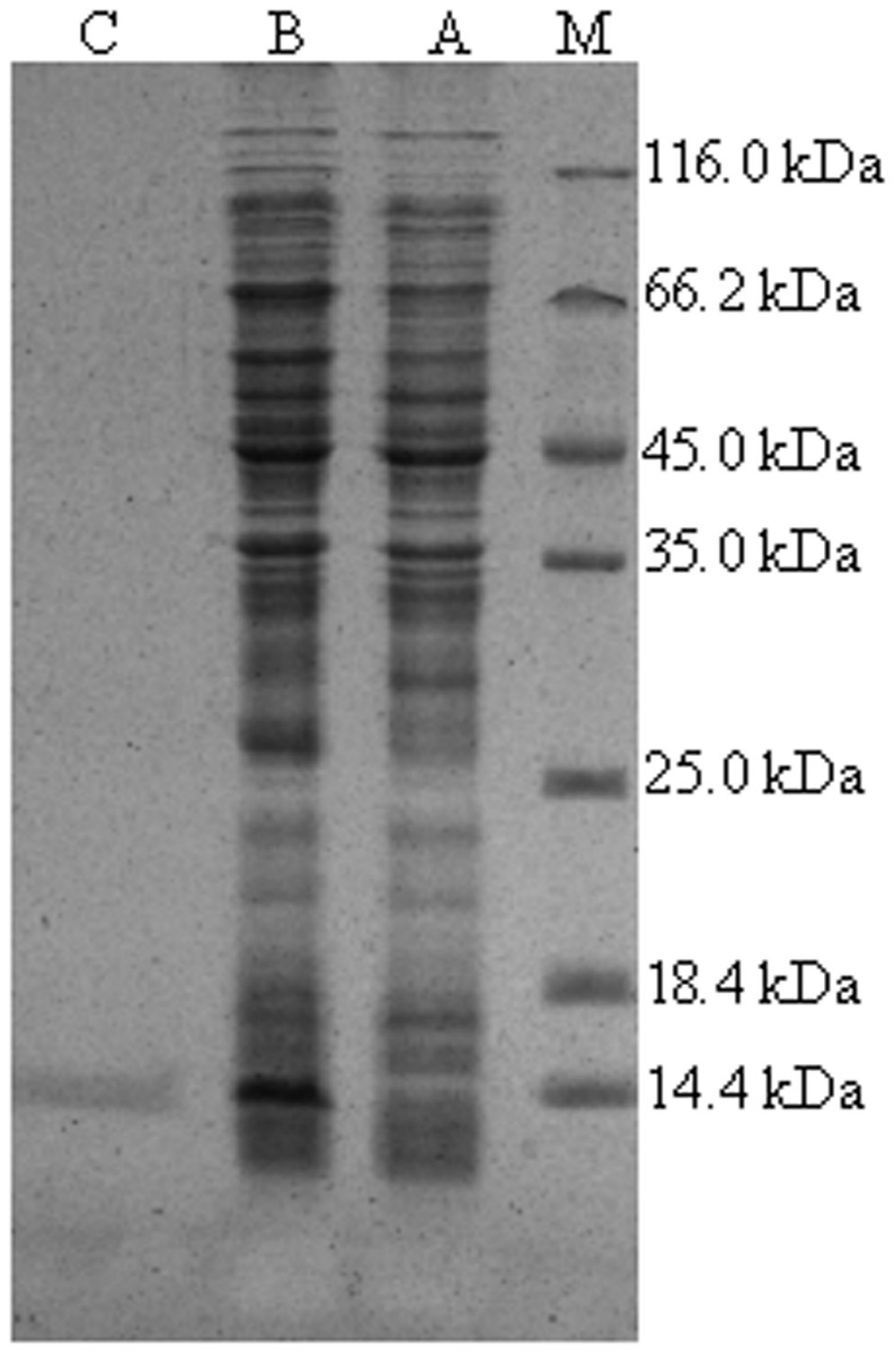
SDS-PAGE analysis of recombinant VpBD. After electrophoresis, the gel was visualized by Coomassie brilliant blue R250 staining. Lane M: protein molecular standard; lane A: negative control for rVpBD (without induction); lane B: induced expression of rVpBD; lane C: purified rVpBD.

The recombinant plasmid pET-21a-VpBD was transformed and expressed in *E. coli* BL21(DE3)-pLysS. After IPTG induction for 3 h, the whole cell lysate analyzed by SDS-PAGE revealed a distinct band with a molecular weight of 9.75 kDa (Fig. 4 lane B), which was further purified to homogeneity by HiTrap Chelating Columns (Fig. 4 lane C). Total of 0.9 mg purified protein was yield from 50 ml bacterial culture in the end.

### MIC assay of the rVpBD

The spectrum of antimicrobial activity of the purified rVpBD was investigated against several Gram-positive and Gram-negative bacteria. The rVpBD could inhibit the growth of all tested microorganisms (Table 1), indicating that rVpBD was a broad-spectrum antibacterial peptide. The highest activity was found against *Staphyloccocus aureus* and *Pseudomonas* with the MIC of 1.64–3.28 µM.

**Table 1:** Antimicrobial activity of rVpBD against Gram-positive and Gram-negative bacteria measured by liquid growth inhibition assay.

Tested microorganisms	MIC value
**Gram-positive bacteria**	
*Staphyloccocus aureus*	1.64–3.28 µM
*Micrococcus luteus*	>26.26 µM
*Bacillus sp.*	13.13–26.26 µM
**Gram-negative bacteria**	
*Vibrio anguillarum*	13.13–26.26 µM
*Entherobacter cloacae*	>26.26 µM
*Vibrio ichthyoenteri*	3.28–6.56 µM
*Pseudomonas putida*	1.64–3.28 µM
*Proteus mirabilis*	>26.26 µM
*Enterobacter sp.*	13.13–26.26 µM

## Discussion

Living in an aquatic environment rich in microorganisms, mollusk has developed effective systems to eliminate noxious microorganisms [16]. However, knowledge advance on the function, expression and regulation of immune effectors in mollusk, especially for AMPs, are still deficient compared with those of insects and vertebrates. In the present study, the cDNA encoding a potential big defensin was identified from *V. philippinarum* (denoted as VpBD). The deduced amino acid of VpBD shared common features of AMPs, such as α-helical structure, net positive charge and high hydrophobic residue ratio. The arrangement of cysteine residues, their neighboring amino acid residues and the spacing between cysteine residues are conserved to previously identified homologues [15], [17], which further indicated that VpBD was a new member of the big defensin family. However, unlike other known big defensin sequences, no prepropeptide sequence was identified from the deduced amino acid of VpBD. The hydrophobic region GAAAVT(A)AA at N-terminus of other counterparts was also absent from VpBD. The above differences and the lower homology between VpBD and other big defensins collectively indicated that VpBD should be a novel big defensin.

Generally, AMPs could be induced by physical stresses and infectious pathogens. The transcript of big defensin from scallop was up-regulated after *V. anguillarum* challenge with a 131.1-fold increase at 32 h compared to the control group [15]. Similar expression pattern was also observed in abalone defensin when received *Vibrios* infection [18]. In contrast, significant decrease of AMPs mRNA expression after *Vibrio* challenge was observed in crustin, mytilin B and penaeidin, respectively [19], [20], [21], [22], [23]. In the present study, the expression level of VpBD mRNA was obviously up-regulated post bacterial challenge, and the peak expression level was detected at 24 hr with a 7.4-fold increase compared with that of control group. The recruitment of VpBD-producing haemocytes into circulating system probably contributed to the drastic increase of VpBD transcript in the early stage of bacterial challenge. After that, the expression level gradually down-regulated, and the lowest expression level was detected at 96 hr post-infection, which was still 3.9-fold higher than the control group. It was postulated that the decreased expression of VpBD was related to the progressive clearance of the invasive pathogens. All these results indicated that VpBD was one of the acute phase proteins involved in the elimination of invasive pathogens.

It is important to understand the microbicidal activities of VpBD *in vitro*. In the present study, the mature peptide of VpBD was expressed in *E. coli* BL21(DE3)-pLysS, and the purified rVpBD exhibited broad-spectrum bactericidal activity towards various bacteria, which was almost consistent with recombinant big defensin from scallop [15] and native protein from horseshoe crab [17]. The same potency and inhibitory effect on growth of Gram-negative (*P. putida*) and Gram-positive bacteria (*S. aureu*s) was also detected for VpBD as big defensin from horseshoe crab [17]. Compared to other molluscan AMPs, the inhibitory activity of VpBD was similar or even more efficient. The MIC of mytilin A towards different bacteria ranged from 0.6 mM to 10 mM [20], [24], while the recombinant defensin from *Crassostrea gigas* showed inhibitory activity towards tested microorganism at µM level [25]. The potent antimicrobial activities of VpBD made it valuable to control outbreak of pathogenic microorganisms in clam culture.

## Materials and Methods

### Clams and bacterial challenge

The clams *V. philippinarum* (7.5–11 g in weight) were purchased from a local market and acclimated for a week before commencement of the experiment. The temperature was held at 20–22°C throughout the whole experiment. The salinity for the supplied seawater was kept at 30‰. For the bacterial challenge experiment, the clams were randomly divided into six flat-bottomed rectangular tanks with 50 liter capacity, each containing 50 clams. One tank served as control, while the other five tanks were immersed with high density of *V. anguillarum* with a final concentration of 10^7^CFU mL^−1^. The infected clams were randomly sampled at 6 hr, 12 hr, 24 hr, 48 hr, 72 hr and 96 hr, respectively. The clams cultured in the normal seawater were used as control group. The haemolymphs from the control and the treated groups were collected using a syringe individually and centrifuged at 2000×*g*, 4°C for 10 min to harvest the haemocytes. There were five replicates for each treatment and the control group.

### cDNA library construction and EST analysis

One clam was randomly selected for cDNA library construction at 8 hr post *V. anguillarum* challenge. The cDNA library was constructed using the ZAP-cDNA synthesis kit and ZAP-cDNA GigapackIII Gold cloning kit (Stratagene). Random sequencing of the library using T3 primer yielded 3226 successful sequencing reactions. BLAST analysis of all the 3226 EST sequences revealed that one EST of 387 bp was highly similar to the previously identified big defensins. Therefore, the EST sequence was selected for further cloning of the full-length cDNA of big defensin from *V. philippinarum*.

### RNA isolation and cDNA synthesis

Total RNA was isolated from the haemocytes of clams using the TRIzol reagent (Invitrogen). First-strand cDNA synthesis was performed according to Promega M-MLV RT Usage information with the RQ1 RNase-Free DNase (Promega)-treated total RNA (1 µg) as template and oligo (dT) primer. The reactions were incubated at 42°C for 1 hr, terminated by heating at 95°C for 5 min. For 5′ RACE, terminal deoxynucleotidyl transferase (Takara) was used to add homopolymer dCTP tails to the 3' end of the purified first-strand cDNA.

### Cloning of the full-length cDNA of VpBD by 5′RACE

Gene-specific primers, P1 and P2 (Table 2), were designed based on the EST to clone the full-sequence cDNA of VpBD. Semi-nested PCR approaches were employed to get the 5′end of VpBD with 1∶50 dilution of the first round PCR products as templates and oligodG as anchored primer. The PCR programs and PCR product sequencing were performed according to previously described [26]. The validity of VpBD cDNA was further verified with primer sets of P3 and P4.

**Table 2:** Primers used in the present study.

Primer	Sequence (5′—3′)	Sequence information
P1(reverse)	ACCTCTACCACTAAGGCATCG	5′ RACE primer
P2(reverse)	CAAATAGCGAATGAGTTAGGAA	5′ RACE primer
P3(forward)	GCTGAATTGAAACTAATTTGACT	Full-length verified primer
P4(reverse)	TTTTTTTTTTTTTTTTTTTTTT	Full-length verified primer
P5(forward)	CTGGTCGCCATGACGACTCTATC	Real time primer
P6(reverse)	CGTTGTCGGGATGGTTCAAGTGC	Real time primer
P7(forward)	CTCCCTTGAGAAGAGCTACGA	Real time actin primer
P8(reverse)	GATACCAGCAGATTCCATACCC	Real time actin primer
P9(forward)	CATATGCTGTGTCTGGACCAAAAGCC	Recombinant primer
P10(reverse)	CTCGAGTTATGGTGGTGGTGGTGGTGGTGACGTCCTGTAATGTG	Recombinant primer

### Sequence analysis of VpBD

The VpBD sequence was analyzed using the BLAST algorithm at NCBI web site (http://www.ncbi.nlm.nih.gov/blast), and the deduced amino acid sequence was analyzed with the Expert Protein Analysis System (http://www.expasy.org/). Sequence alignment of VpBD was performed with the ClustalW Multiple Alignment program (http://www.ebi.ac.uk/clustalw/) and Multiple Alignment show program (http://www.biosoft.net/sms/index.html).

### mRNA expression profile of VpBD post *V. anguillarum* challenge

The expression of VpBD transcript in haemocytes after *Vibrio* challenge was measured by quantitative real time RT-PCR in Applied Biosystem 7500 fast Real-time PCR System. Gene-specific primers P5 and P6 (Table 2) were designed to amplify a PCR product of 101 bp. The product was purified and sequenced to verify the PCR specificity. Two clam β-actin primers, P7 and P8 (Table 2) were used to amplify a 121 bp fragment as internal control to verify the successful reverse transcription and to calibrate the cDNA template. The reaction component, thermal profile, and the data analysis were conducted as previously described [26]. All data were given in terms of relative mRNA expression as means ± S.E. The results were subjected to One-way Analysis of Variance (ANOVA) to determine differences in the mean values among the treatments. Significance was concluded at *P*<0.05. Statistical analysis was performed using SPSS 11.5 for Windows.

### Recombinant expression of VpBD and protein purification

PCR fragment encoding the mature peptide of VpBD was amplified with gene-specific primers P9 and P10 with Nde I and Xho I sites at their 5′ end, respectively (Table 2). The PCR product was cloned into pMD18-T simple vector (Takara), digested completely by restriction enzymes Nde I and Xho I (NEB), and then subcloned into the Nde I/Xho I sites of expression vector pET-21a(+) (Novagen). The recombinant plasmid (pET-21a-VpBD) was transformed into *Escherichia coli* BL21 (DE3)-plysS (Novagen) and subjected to DNA sequencing. After sequencing to ensure in-frame insertion, positive clones were incubated in SOB medium (containing 50 mg/L ampicillin) at 37°C with shaking at 220 rpm. When the culture reached OD_600_ of 0.6, IPTG with final concentration of 1 mmol/L was added to the culture, and incubated for additional 3 hr under the same conditions. Cells were harvested by centrifugation at 10,000 g for 2 min, and suspended in 50 mM Tris containing 5 mM EDTA, 50 mM NaCl, and 5% Glycerol (pH 7.9). After being sonicated at 4°C for 60 min, the rVpBD was purified by HisTrap Chelating Columns (Amersham Biosciences) according to the manufacturer's instruction. The purified protein was subjected to 15% SDS-PAGE according to the method of Laemmli [27]. The concentration of rVpBD was measured by BCA Protein Assay Kit.

### Antimicrobial activity of rVpBD

Antibacterial testing was carried out using three Gram-positive bacteria (*Staphyloccocus aureus, Micrococcus luteus* and *Bacillus sp*) and six Gram-negative bacteria (*Vibrio anguillarum*, *Entherobacter cloacae, Pseudomonas putida, Proteus mirabilis*, *Vibrio ichthyoenteri* and *Enterobacter sp*.). The MIC was determined according to the method of Hancock (http://cmdr.ubc.ca/bobh/methods/). The assay was done with triplicates in three independent experiments. The MIC value was recorded as the range between the highest concentration of the protein where bacterial growth was observed and the lowest concentration that caused 100% inhibition of bacteria growth.
